# Risk Factors Associated with the Emergence of Multidrug-Resistant Bacteria and Fungal Infections in Walled-Off Pancreatic Necrosis

**DOI:** 10.3390/antibiotics15020220

**Published:** 2026-02-17

**Authors:** Michael Fernandez Y Viesca, Alia Hadefi, Lukas Otero Sanchez, Martina Pezzullo, Morgane Van Wettere, Eleni Karakike, Maya Hites, Viviane De Maertelaer, Myriam Delhaye, Marianna Arvanitakis

**Affiliations:** 1 Department of Gastroenterology, Hepatopancreatology and Digestive Oncology, Hôpital Universitaire de Bruxelles, Université Libre de Bruxelles, 1070 Brussels, Belgium; alia.hadefi@hubruxelles.be (A.H.); lukas.oterosanchez@hubruxelles.be (L.O.S.); myriam.delhaye@hubruxelles.be (M.D.); marianna.arvanitaki@hubruxelles.be (M.A.); 2 Department of Radiology, Hôpital Universitaire de Bruxelles, Université Libre de Bruxelles, 1070 Brussels, Belgiummorgane.vanwettere@hubruxelles.be (M.V.W.); 3 Department of Critical Care, School of Nursing, National and Kapodistrian University of Athens, 124 62 Athens, Greece; 4 Clinic of Infectious Diseases, Hôpital Universitaire de Bruxelles, Université Libre de Bruxelles, 1070 Brussels, Belgium; maya.hites@hubruxelles.be; 5 Institut de Recherche en Biologie Humaine et Moleculaire, Université Libre de Bruxelles, 1070 Brussels, Belgium; viviane.de.maertelaer@ulb.be

**Keywords:** acute necrotizing pancreatitis, infected pancreatic necrosis, drug resistance, multiple bacterial infections, antibiotics, antifungal therapy, collection drainage, walled-off necrosis

## Abstract

Background: Infected pancreatic necrosis (IPN) is a serious complication of moderate-to-severe acute pancreatitis (AP), associated with high morbidity, intensive care unit (ICU) admission, organ failure, and mortality. Initial management relies on antibiotics and drainage of walled-off necrosis (WON). In the context of increasing multidrug-resistant (MDR) bacteria, identifying risk factors for MDR emergence is crucial. The impact of fungal infections (FIs) on outcomes also remains unclear. This study aimed to identify risk factors associated with the emergence of MDR bacteria and FIs during intervention for IPN. Methods: This retrospective study included 71 consecutive patients undergoing intervention for suspected IPN or symptomatic WON. Results: At first intervention, IPN was confirmed in 52 patients (73%), MDR bacteria in 19 (27%), extensively drug-resistant (XDR) bacteria in 4 (5.6%), and FI in 21 (30%). After all interventions, MDR/XDR bacteria and fungi were detected in 25 (35%)/11 (15.5%) and 42 (59%) patients, respectively. Independent risk factors for MDR emergence were the number of antibiotic changes (b, 1.70; 95% CI 1.18–2.43; *p* = 0.004) and need for nutritional support (NS) (b, 5.69; 95% CI 1.52–20.50; *p* = 0.010). No independent factor was associated with FI. The 180-day mortality did not differ across groups. The 90-day cumulative ICU admission rate was higher in IPN vs. non-IPN (63.1% vs. 29.4%, *p* = 0.030) and in MDR vs. non-MDR (72.2% vs. 37.1%, *p* = 0.005). Conclusions: Antibiotic changes and NS were independently associated with MDR emergence in IPN. No independent factors were linked to FI. ICU admission was significantly higher in IPN and MDR cases.

**Key Summary**:

**Established knowledge of the subject**

° Infected pancreatic necrosis is a serious complication related to acute necrotizing pancreatitis and is related to high morbidity and mortality.
° Antibiotic treatment combined with drainage/debridement through mini-invasive interventions is a key step in therapeutic management.
° Although broad-spectrum antibiotics are often required as first-line therapy in suspected infected pancreatic necrosis, prolonged empirical use without microbiological guidance may contribute to the emergence of multidrug-resistant organisms.

**Significant and/or new findings of this study**

° This retrospective study included 71 patients with acute necrotizing pancreatitis, infected pancreatic necrosis (IPN), and walled-off necrosis (WON) having undergone minimally invasive interventions (endoscopy and radiology) for drainage (+/− debridement) with initial and subsequent documented bacteriological culture from the WON.
° We revealed an increase in bacterial resistance during the treatment period: multidrug-resistant bacteria were already present at the first intervention in 27% of patients, extensively drug-resistant bacteria in 5.6%, and fungal infection in 30%, increasing to 35%, 15.5% and 59%, respectively, at the end of the treatment period.
° Antibiotic changes and the need for nutritional support were independently associated with the emergence of multidrug-resistant bacteria in patients with IPN.
° These findings support the use of antimicrobial stewardship initiatives aiming to rationalize antibiotic use and emphasize shorter, more targeted antibiotic therapy for patients with IPN.

## 1. Introduction

Acute pancreatitis (AP) is a common condition worldwide, and most patients have a rapidly favorable outcome [1,2]. However, 20% of patients will develop moderate-to-severe disease associated with the presence of necrotic pancreatic tissue [3]. These patients have a high risk of necrotic infection (up to 70%) [4]; two-thirds will need intervention [5], one-third will develop organ failure [6], and 15%–20% will die [7]. Infected pancreatic necrosis (IPN) results from disruption of the intestinal mucosal barrier, leading to increased permeability and bacterial/fungal translocation into pancreatic and peripancreatic necrotic tissue [8]. According to international guidelines, IPN requires management with antibiotics and minimally invasive interventions, including endoscopic and radiological drainage of collections (walled-off necrosis (WON)) [9,10,11]. Antibiotics play an important role in the conservative management of suspected and confirmed IPN [5]. However, the efficacy of previous antibiotic therapy for sterilizing infected collections or avoiding intervention is not well known [5,12]. Moreover, there are no clear recommendations concerning the type or duration of first-line antibiotic therapy nor the use of concomitant antifungal therapy [9,10,11]. In the era of multidrug-resistant bacteria (MDR) due to antibiotic overuse, microbiological data are of significant interest [13,14]. Also, although they are less frequent, the impact of fungal infections (FIs) on clinical outcomes has recently been highlighted, with reported incidences ranging from 7 to 46% in previous studies [15,16].

This study aimed to identify risk factors associated with the emergence of MDR bacteria during minimally invasive interventions for IPN and to identify factors associated with FI during the initial intervention for IPN. Secondary endpoints were to identify overall risk factors for IPN and to describe our local microbial epidemiology; the antibiotic therapy administered; and the natural course of selection towards MDR, extensively drug-resistant (XDR), and pan-drug-resistant (PDR) bacteria and their impact on disease outcomes (mortality and ICU admission rates).

## 2. Results

### 2.1. Characteristics of the Population

**Figure 1:**
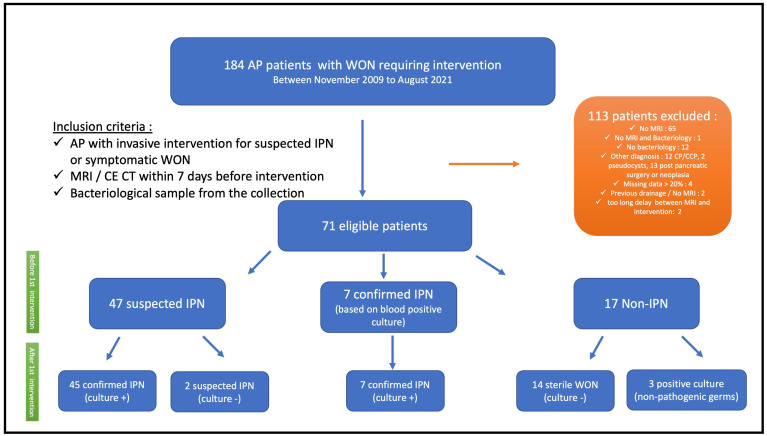
Flowchart of the population. In total, 184 AP patients with WON requiring intervention were considered for this study. Of these, 113 patients who did not fulfill the inclusion criteria were excluded, bringing the total number of included patients to 71. Among them, 52 patients had confirmed IPN, 2 patients had suspected IPN, and 17 had non-IPN. The definition of confirmed IPN was a patient with a positive culture of fluid/necrosis collected during the first intervention or, in the case of positive blood cultures, if there was no other documented extra-pancreatic infection (40). The definition of suspected IPN was considered for one of the following situations: sustained septic shock, sepsis, or SIRS (at least 2 criteria); persistent (>3–5 days) or new onset of fever and elevated CRP levels and WBC without any other documented extra-pancreatic infection; imaging suggestive of infection by CE-CT (bubble gas) (41) or MRI with DWI (presence of thick wall and restriction of diffusion) (39); or clinical and imaging features consistent with IPN despite negative cultures. Non-IPN was defined by the absence of any pathogenic microorganism in the culture obtained during the initial intervention, along with no clinical and radiological evidence of infection. A sterile collection was defined by the absence of any microorganism in the culture obtained during the initial intervention. Abbreviations: AP: acute pancreatitis, IPN: Infected pancreatic necrosis, CE-CT: contrast-enhanced computed tomography, CP: chronic pancreatitis, CCP: chronic calcifying pancreatitis, DWI: diffusion-weighted imaging, MRI: magnetic resonance imaging, SIRS: systemic inflammatory response syndrome, WON: walled-off necrosis.

From November 2009 to August 2021, 184 patients with WONs requiring intervention for suspected IPN or other indications (e.g., pain and organ compression) were assessed for eligibility, and 71 patients were included in the study (Figure 1). The mean age was 51 ± 16 years, and the majority of patients were men (72%). Alcohol abuse and gallstones were the leading causes of AP in 38% and 33%, respectively. According to the Atlanta Classification, 45% were considered severe and 55% moderate. All patients underwent a first intervention with a median of 36 days (24–69) from the onset of symptoms, and endoscopic drainage was performed in all 71 patients. After the first intervention, fifty-two patients (73%) were considered to have confirmed IPN, including 7 patients with positive blood cultures without any other documented extra-pancreatic infection and 45 patients with positive cultures of fluid/necrosis collected during the first intervention. Two patients had suspected IPN (clinic/imaging suggestive of IPN but first culture negative), and seventeen patients (24%) had non-IPN (Figure 1). The characteristics of the included patients are detailed in Table 1.

### 2.2. Microbiological Culture and Antibiotics Before and After Intervention

Table 2 summarizes the results of microbiological and antibiotic characteristics of the population before the first intervention. In 56 patients (79%), antibiotics had been initiated for a median duration of 7 days (2–18) prior to the first drainage (92 periods of antibiotics: 67 cases (73%) were treated empirically and 25 (27%) based on a positive culture (e.g., blood culture)). Among these 56 patients, 29 patients (52%) and 27 patients (48%) received one or more than one antibiotic, respectively. Thirty-one patients (44%) were free from antibiotics for at least seven days before the first intervention. Of the remaining 40 patients who received antibiotics within seven days before the first intervention, five patients (12.5%) had sterile collections. The most frequently administered antimicrobial category was antipseudomonal penicillin + ß-lactamase inhibitor (33%), followed by penicillin + ß-lactamase inhibitor (28%) and carbapenem (15%). Other combinations of different classes of antibiotics are detailed in Table 2. Six patients received concomitant antifungal therapy before the first intervention, of whom one patient showed fungi in the culture obtained during the initial intervention.

**Figure 2:**
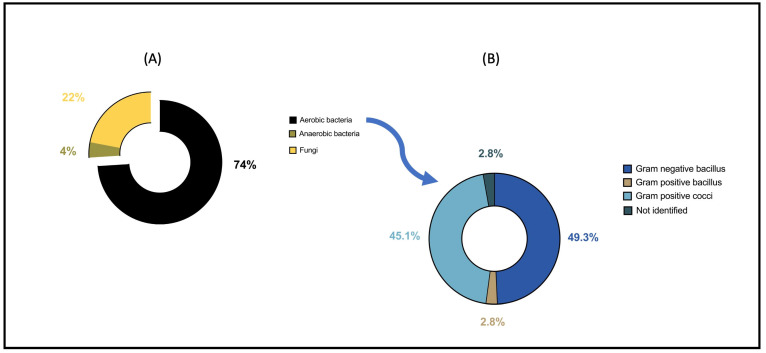
Microbiological culture results from the first intervention. Proportion of aerobic, anaerobic, and fungal organisms (**A**) and classification of aerobic bacteria (**B**) isolated from the first sample obtained at the time of initial drainage of the WON. Abbreviations: WON: walled-off necrosis.

Figure 2A,B and Supplementary Table S3 show the different microbiological agents isolated from the sample at the time of the first intervention. Among aerobic bacteria, Gram-negative bacilli (GNB) and Gram-positive cocci (GPC) represented 49% and 45% of the isolates, respectively. Among anaerobes, five strains were isolated (*Fusobacterium nucleatum* n = 2, *Lactobacillus gasseri* n = 1, *Prevotella buccae* n = 1, and *Veillonella parvula* n = 1). Fungi were isolated in 21 patients, mainly *Candida albicans* (n = 17, 81%), followed by *Candida glabrata* (n = 3, 14%) and *Candida krusei* (n = 1, 5%). Regarding antifungal susceptibility, all *Candida albicans* isolates were susceptible to fluconazole, except for one strain that was resistant to fluconazole and other triazoles (itraconazole, voriconazole, and posaconazole) but remained susceptible to caspofungin. Two of the three *Candida glabrata* isolates showed intermediate susceptibility to fluconazole but were susceptible to other antifungal classes. The *Candida krusei* isolate was resistant to fluconazole and itraconazole.

After the first intervention, all but one (99%) patient received antibiotics. Initial treatment mainly consisted of penicillin + ß-lactamase inhibitors (34%), antipseudomonal penicillin + ß-lactamase inhibitors (31%), and carbapenem (17%). The first-line antibiotic regimen was adequate according to bacterial susceptibility in 51/70 patients (73%). The only patient who did not receive antibiotics at the time of the first drainage had a negative culture. During follow-up, antifungal therapy was administered in 31/70 patients (44%) and always in combination with antibiotics. Antibiotic regimens were frequently modified according to microbiological results and clinical evolution, with a median of 2 adaptations (range 1–4) per patient. Overall, patients received a median of 4 (2–6) different antimicrobial agents, with a mean total duration of antibiotic therapy of 43 ± 30 days. These results are summarized in Table 3.

### 2.3. Risk Factors Associated with Emerging MDR

**Figure 3:**
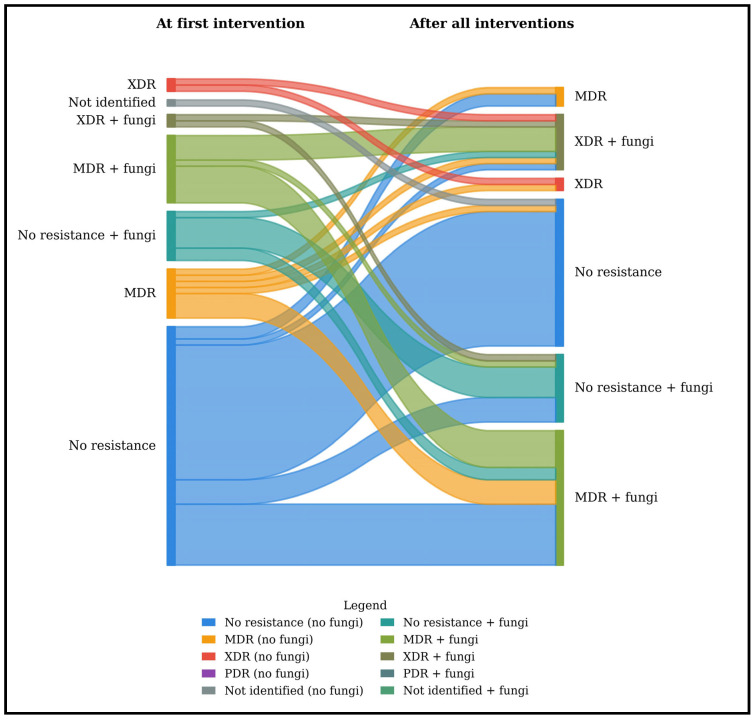
Dynamics of bacterial resistance and fungal detection at the time of the first and following the different interventions. Sankey diagram illustrating changes in bacterial resistance profiles and fungal co-infection status at first intervention and after all interventions among 71 patients. At first intervention, MDR and XDR bacteria accounted for 26.8% and 5.6% of isolates, respectively, increasing to 35.2% and 15.5% during follow-up, while no PDR bacteria were detected. The proportion of patients with fungal detection increased from 29.6% to 59.2%. According to the in vitro antimicrobial susceptibility testing, each germ was classified as follows: No Resistance: non-susceptible to < 1 agent in < 3 antimicrobial categories; MDR: non-susceptible to ≥1 agent in ≥3 antimicrobial categories; XDR: non-susceptible to ≥1 agent in all but ≤2 categories; PDR: non-susceptible to all antimicrobial agents listed (42). Abbreviations: MDR: multidrug-resistant bacteria, PDR: pan-drug-resistant bacteria, XDR: extensively drug-resistant.

Figure 3 summarizes the distribution of bacterial resistance profiles at the first intervention and during follow-up in patients with WON. At the first intervention, the proportion of MDR and XDR bacteria was 26.8% and 5.6%, respectively, and this rose to 35.2% and 15.5%, respectively, during the follow-up intervention. No PDR bacteria were observed in this cohort. In logistic regression, risk factors associated with the emergence of MDR bacteria during the natural course of AP were analyzed (Table 4). In univariate analysis, prior exposure to antibiotics, the number of changes in antibiotics, ICU admission, size of the WON (length and width), the need for NS, AP duration, albumin levels, and total number of interventions ≥ 5 were associated with the emergence of MDR during the natural course of the disease. In multivariate analysis, only the number of changes in antibiotics (b, 1.70; 95% CI, 1.18–2.43; *p* = 0.004) and the need for NS (b, 5.69; 95% CI, 1.52–20.50; *p* = 0.010) were independently associated with the emergence of MDR bacteria.

### 2.4. Risk Factors Associated with FI

Figure 3 also presents the dynamics of fungal detection during the primary intervention and thereafter, during the course of the disease. Of the 21 patients identified to have an FI during the first intervention, four (19%) were resistant to fluconazole. A logistic regression to determine potential risk factors associated with FI at the first intervention was performed (Table 5). In univariate analysis, only the total number of interventions was significantly associated with FI (OR, 3.43; 95% CI, 1.2–10.22; *p* = 0.023).

Among the 42 patients with positive fungal cultures after all interventions, antifungal therapy was administered to 26 (62%), whereas five patients were treated while no fungi were detected.

### 2.5. Risk Factors Associated with Overall IPN

After a logistic regression, the two independent factors associated with the overall occurrence of IPN were prior exposure to antibiotics (b, 26.41; 95% CI, 1.90–366.38; *p* = 0.015) and prealbumin levels (b, 0.77; 95% CI, 0.63–0.94; *p* = 0.012) (Table 6)

### 2.6. Mortality and ICU Admission Rate Analysis

**Figure 4:**
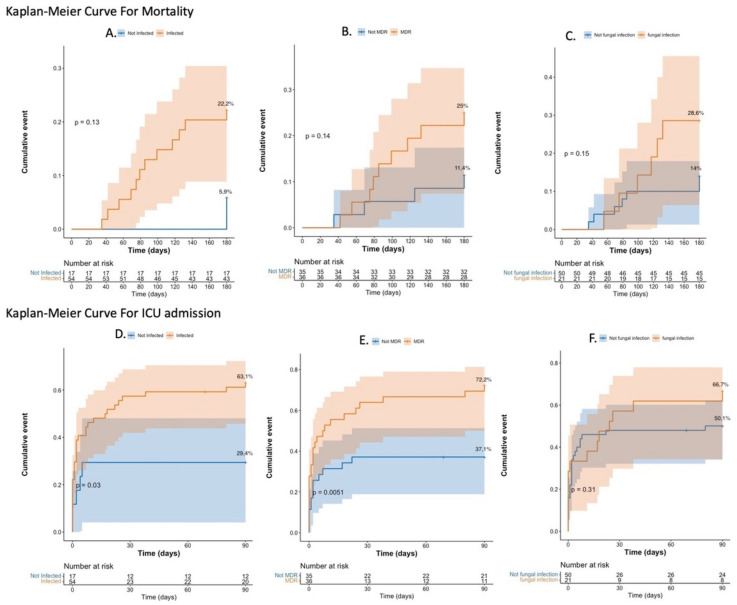
Kaplan–Meier curves for mortality (**A**–**C**) and ICU admission (**D**–**F**). (**A**,**D**) IPN versus non-IPN; (**B**,**E**) MDR versus non-MDR bacteria; (**C**,**F**) FI versus not FI. Abbreviations: ICU: intensive care unit; IPN: infected pancreatic necrosis; MDR: multidrug-resistant; PFI: primary fungal infection.

The observed mortality rate in this cohort was 18% (13 patients), all but one of them with IPN. The 180-day mortality rate (Figure 4A–C) was not significantly different between IPN and non-IPN patients: 22.2% (95% CI, 10.3%–32.6%) vs. 5.9% (95% CI, 0%–16.4%), *p* = 0.130; between MDR and non-MDR: 25% (95% CI, 9.4%–37.9%) vs. 11.4% (95% CI, 0.24%–21.4%), *p* = 0.140; and between FI and non-FI (at the first drainage): 28.6% (95% CI, 6.4%-45.5%) vs. 14% (95% CI, 3.8%–23.1%), *p* = 0.150. The 90-day cumulative ICU admission rate (Figure 4D–F) was significantly different between the IPN and non-IPN groups: 63.1% (95% CI, 47.7%–74%) vs. 29.4% (95% CI, 4.1%–48.1%), *p* = 0.030, and between MDR and non-MDR: 72.2% (95% CI, 53%–83.6%) vs. 37.1% (95% CI, 19%–51.3%), *p* = 0.005, but not significant between FI and non-FI (at the first drainage): 66.7% (95% CI, 39%–81.8%) vs. 50.1% (95% CI, 34.1%–62.2%), *p* = 0.310.

## 3. Discussion

To our knowledge, this is the first study to demonstrate a dynamic progression of the emergence of MDR bacteria throughout the course of subsequent interventions in IPN patients. Unlike previous studies that provide only a single-timepoint snapshot of MDR prevalence [14,17,18,19,20], these data show a clear temporal evolution: the incidence of MDR and XDR in IPN increased from 26.8% to 35.2% and from 5.6% to 15.5%, respectively, between the first and the final interventions.

These high incidences remain comparable to those reported in the most recent retrospective studies, where MDR rates range from 29.5% to 57.5% [14,17,18,19,20]. One explanation for these high incidence rates could be that these patients were treated in a tertiary center, specialized in treating this type of severe AP that requires multidisciplinary care. Another explanation could be that the severity of disease in our population was greater than that of other published cohorts, supported by the cumulative ICU admission rates, which were significantly higher in patients with IPN and MDR bacteria than in those with non-IPN and non-MDR bacteria. Moreover, ICU settings themselves may contribute to the emergence of MDR organisms due to factors such as prolonged antibiotic exposure, invasive procedures, and increased selective pressure in critically ill patients [18].

Although this study did not demonstrate any statistically significant difference in mortality between patients with and without MDR bacteria, as in the aforementioned studies [14,17,18,19,20,21], there was, nonetheless, a trend toward higher mortality in the MDR group.

Two independent risk factors associated with the emergence of MDR bacteria in IPN were identified—the number of antibiotic changes and the need for NS (enteral and/or parenteral nutrition)—whereas no independent risk factors were identified regarding the occurrence of FI at the first intervention.

In 2022, Lu et al. published a retrospective study analyzing 124 MDR and 143 non-MDR bacterial isolates from patients with IPN and identified the presence of extra-pancreatic infections, procalcitonin (PCT) level at admission, and the degree of pancreatic necrosis as independent risk factors for MDR bacteria isolation in IPN [14]. In contrast to our study, none of the patients were treated endoscopically, which does not represent current practice [4].

The results of this study highlight the importance of antibiotic selective pressure on the emergence of MDR pathogens [13,14] and preventing and treating malnutrition during ANP to avoid infection [22,23]. Regarding antibiotic pressure, Boxhoorn et al. showed in a recent randomized study that there was no advantage to immediate drainage in patients with suspected IPN compared to initial treatment with antibiotic therapy and postponed drainage, after which a third of patients improved with antibiotics alone [5]. However, targeted treatment could reduce the selective pressure of antibiotics on the emergence of resistant strains [14]. It is worth noting that patients with MDR and XDR infections required the greatest number of antibiotic changes, as treatments had to be continuously adapted according to microbiological culture results. This raises the question of causality—whether repeated antibiotic modifications contribute to the selection of resistance or, conversely, simply reflect the presence of already resistant infections. Moreover, the potential role of antibiotic-related toxicity, which may have prompted therapy changes in certain cases, should not be overlooked, as it could also have influenced the overall number of antibiotic switches [24]. These findings may support a more cautious and individualized approach to antibiotic therapy, potentially favoring early discontinuation when WONs are well drained and patients are hemodynamically stable, with only limited peri-interventional antibiotic use, and the acquisition of bacteriological documentation during interventions.

Regarding the nutritional aspect, several meta-analyses have shown that NS is a cornerstone of managing patients with AP [25,26]. Enteral nutrition (EN) is superior to parenteral nutrition (PN) in reducing mortality, persistent organ dysfunction, and systemic infection, as well as preserving mucosal integrity and intestinal motility [25,26], and therefore preventing bacterial translocation and secondary IPN [27]. In this study, NS was necessary in almost two-thirds of patients. The fact that the need for NS was identified as an independent risk factor for the emergence of MDR in IPN probably reflects a more severe and prolonged course of the disease related to malnutrition requiring NS and not a direct effect of NS.

Although the rate of FI doubled between the first intervention and the last one, our study did not identify any independent risk factors associated with FI at the first intervention. The decision to investigate risk factors of FI only at the first intervention, rather than across all interventions, was based on the fact that no previous studies have examined risk factors of FI at the time of the initial drainage, which can be more of a surrogate marker for overall disease severity. Indeed, the absence of a mortality difference between patients with and without FI at the first intervention indicates that FI may represent a marker of disease progression rather than a direct determinant of outcome. These findings are consistent with recent data from the United States supporting the idea that FI in AP may emerge as secondary events rather than primary complications [15]. Unlike some previous studies, prior antibiotic exposure did not appear to play a decisive role [28]. The variability in antifungal therapy observed across studies underlines the current lack of consensus regarding treatment indications in this setting [4,15]. Taken together, our results emphasize the need for clearer diagnostic criteria and evidence-based therapeutic guidelines for managing FI in IPN.

From an epidemiological perspective, this study showed that aerobic bacteria are predominant compared to anaerobic bacteria, as previously reported [4,29], with GNB and GPC the most represented [30,31,32]. From an antibiotic standpoint, the main classes administered before and after the initial intervention were predominantly penicillin + ß-lactamase inhibitors, antipseudomonal penicillin + ß-lactamase inhibitors, and carbapenem. Although robust evidence to guide empirical antibiotic selection is lacking [9,10,11], agents with good penetration into pancreatic necrosis are generally recommended. These include broad-spectrum penicillins, third- and fourth-generation cephalosporins, carbapenems, or fluoroquinolones with anaerobic coverage such as metronidazole [29,33,34,35,36]. Although the duration of antibiotics may appear prolonged in this study (43 days), current evidence does not clearly define the optimal length of antibiotic treatment in patients with IPN undergoing multiple drainage or necrosectomy procedures [13]. Ongoing antimicrobial stewardship initiatives aim to rationalize antibiotic use, and it is likely that future recommendations will emphasize shorter, more targeted therapy for IPN.

Our study has some limitations: First, it is retrospective and monocentric and has a limited number of patients. Second, this study was carried out in a tertiary center that is referenced for the treatment of complex cases; therefore, it does not fully represent all hospital settings. Moreover, we did not study the role of PCT, as we do not routinely measure it in daily practice; nevertheless, it could be a useful parameter to diagnose IPN by helping to differentiate patients who need antibiotics from those who present with an AP-related inflammatory reaction, especially during the first period of the disease [37]. Finally, the absence of independent risk factors for FI may be explained by the limited number of cases and the low proportion of immunocompromised patients in our cohort, which reduced the statistical power of the analysis.

Further studies should focus on improving the diagnosis of IPN, specifically using DWI-MRI and PCT to optimize when to initiate antibiotic therapy. This approach may help to better identify patients who need minimally invasive interventions with subsequent use of pathogen-directed antibiotics and thereby prevent the emergence of increasingly resistant bacteria. Another issue is determining the type and duration of antibiotic and antifungal therapy and how and when to prescribe them while limiting their duration.

## 4. Materials and Methods

### 4.1. Study Population

This was a retrospective study that included data on seventy-one patients with moderate-to-severe acute pancreatitis according to the Atlanta Classification 2012 [38], admitted between November 2009 and August 2021 to HUB-Erasme Hospital in Brussels. All consecutive patients who underwent a minimally invasive intervention for WON following ANP (for suspicion of infection, pain or extrinsic compression of surrounded organs) with a documented bacteriological culture from the WON and both contrast-enhanced computed tomography scan (CE-CT scan) and magnetic resonance imaging (MRI), including diffusion-weighted (DW) acquisition within a maximum of seven days before the first intervention, were included. Exclusion criteria were as follows: the presence of chronic pancreatitis, no CE-CT, MRI without specific DW-MRI sequences before the first intervention, or no bacteriological culture performed during the first intervention. MRI with DWI was considered a prerequisite for inclusion due to the concomitant goal to assess the diagnostic yield of DWI to confirm the presence of IPN [39], which will be reported in a separate study. Collected data for each patient are presented in Supplementary Table S1. The last follow-up was defined as either the date of the last documented clinical encounter (e.g., outpatient consultation) or the date of death.

### 4.2. Definitions

The patient was classified as having confirmed IPN if they had a positive culture of fluid/necrosis collected during the first drainage intervention for the WON or, in the case of positive blood cultures, without any other documented extra-pancreatic infection [40].

The patient was classified as having suspected IPN in the following situations: sustained septic shock, sepsis, or SIRS (at least 2 criteria); persistent (>3–5 days) or new onset of fever and elevated CRP levels and WBC without any other documented extra-pancreatic infection; imaging suggestive of infection by CE-CT (gas configurations within the WON) [41] or MRI with DWI (presence of thick wall and restriction of diffusion) [39]; or clinical and imaging features consistent with IPN despite negative cultures.

Non-IPN was defined by the absence of any pathogenic microorganism in the culture obtained during the initial intervention, along with no clinical or radiological evidence of infection. A sterile collection was defined by the absence of any microorganism in the culture obtained during the initial intervention.

FI was defined by the presence of fungi in the WON culture obtained during the first or subsequent interventions.

### 4.3. Microbiological Cultures and Antibiotics

Microbiological data collected for each patient, as well as definitions regarding in vitro antimicrobial susceptibility testing, are presented in Supplementary Table S2 [42].

### 4.4. Primary Endpoint

The primary endpoint was to identify risk factors associated with the emergence of MDR bacteria (aerobes/anaerobes) and FI (at the first drainage) in IPN.

### 4.5. Secondary Endpoints

Secondary endpoints included defining risk factors of overall IPN (including non-MDR, MDR, XDR, and PDR bacteria), characterizing local epidemiology of bacterial isolates from WONs, reporting the antibiotic and antifungal therapy administered, assessing adequacy of the first antibiotic choice based on the first bacteriological culture (defined as the in vitro susceptibility of the microorganism(s) isolated from the first positive culture to the initial empiric antibiotic regimen administered), and comparing survival and cumulative ICU admission rates between patients with IPN/non-IPN, MDR/non-MDR bacteria, and FI/non-FI.

### 4.6. Statistical Analysis

Continuous variables are described as means ± standard deviation (SD) or medians and interquartile range (IQR) in cases of non-normally distributed variables. Categorical variables are reported as counts and percentages. A logistic dichotomous dependent variable was used to identify factors associated with the emergence of MDR bacteria, FI at the first drainage, and IPN. A multivariable model was subsequently built using a stepwise procedure by introducing to the multivariate analyses only factors that reached significance in the univariate analysis.

The Kaplan–Meier approach was used to assess the survival rate incidence at 180 days and the cumulative rate of ICU admission at 90 days following AP diagnosis.

Data for patients were censored at 180 days for mortality or at 90 days for ICU admission.

A *p*-value < 0.05 was required for significance for the multivariable model.

Statistical analyses were performed using SPSS for macOS (version 28) and R for macOS (version 2023.06.1 + 524). Data processing and visualization, including the generation of Sankey diagrams, were performed using Python (version 3.11) with the pandas and matplotlib libraries.

## 5. Conclusions

The emergence of MDR bacteria in patients with IPN who required invasive procedures was observed in an alarming proportion of this cohort (51% of patients with MDR or XDR bacteria). The emergence of MDR bacteria was related to the number of antibiotic changes and the need for NS. In contrast, no independent factors for FI at first drainage were identified. Although this study did not demonstrate a significant difference in mortality between MDR and non-MDR IPN, the cumulative ICU admission rate was significantly higher in the MDR group.

## Figures and Tables

**Table 1:** Characteristics of included patients.

Characteristic	WON Acute Pancreatitis Cohort (N = 71)
Age, years [95% CI]	51 ± 16 [48–55]
Male sex, n (%)	51 (72)
Cause of pancreatitis, n (%)	
- Alcohol abuse - Gallstones - Idiopathic - Post-ERCP - Traumatic - Autoimmune	27 (38) 23 (33) 15 (21) 3 (4) 2 (3) 1 (1)
Disease severity, n (%)	
- Revised Atlanta Classification: moderate/severe - Admission to ICU - Systemic inflammatory response syndrome (>2 criteria) - Organ failure ^†^ - Multiple organ failure ^†^ - Persistent organ failure (>48 h) ^†^	39(55)/32(45) 39 (55) 21 (30) 38 (56) 26 (38) 29 (43)
CT Severity Index score on admission, n (%) ^‡^	6 ± 3 [5–7]
Extent of pancreatic necrosis on admission, n (%) ^‡^	
- <30% - 30 to 50% - >50%	41 (59) 13 (19) 15 (22)
Diabetes, n (%)	20 (28.2)
Body mass index, kg/m^2^ [95% CI]	28 ± 5 [26–29]
Proton-pump inhibitor therapy, n (%)	65 (91.5)
Diagnosis of infected pancreatic necrosis ¶, n (%)	54 (76)
Interventions related to infected pancreatic necrosis, n (%)	
- Endoscopic drainage - Endoscopic necrosectomy - Percutaneous drainage - Surgical drainage - VARD	71 (100) 25 (35) 14 (20) 10 (14) 4 (6)
No. of days from onset of symptoms to diagnosis of ANP ^§^, days, median (IQR)	5 (2–8)
No. of days from onset of symptoms to diagnosis of IPN, days, median (IQR)	36 (24–69)
Nutritional support, n (%)	46 (65)
- Enteral - Parenteral - Mixed Enteral/Parenteral	15 (21) 10 (14) 21 (30)
Length of stay, days, median (IQR)	58 (41–91)
Mortality, n (%)	13 (18)

ANP: acute necrotizing pancreatitis; CI: confidence interval; ERCP: endoscopic retrograde cholangiopancreatography; ICU: intensive care unit; IQR: interquartile range; IPN: Infected pancreatic necrosis, VARD: videoscopic assisted retroperitoneal debridement. ^†^ Based on 68 patients (3 cases of missing data); ^‡^ based on 69 patients (2 cases of missing data) and performed at least 48–72 h after the onset of symptoms; ¶ Confirmed (n = 52) and suspected (n = 2) IPN; ^§^: based on CE-CT. Quantitative data are expressed as mean ± standard [IC 95%] unless otherwise indicated.

**Table 2:** Microbiological characteristics of the patients before ^†^ the first intervention.

Characteristic	WON Acute Pancreatitis Cohort (N = 71)
Prior exposure to antibiotics, n (%)	56 (79)
No. of prior antibiotics, n (%)	
- 0 - 1 - ≥1	15 (21) 29 (41) 27 (38)
Prior antifungal therapy, n (%) ^‡^	6 (8.5)
Type of antibiotic therapy before the first intervention, n (%) ^§^,	
- Penicillin + ß-lactamase inhibitor - Antipseudomonal penicillin + ß-lactamase inhibitor - Carbapenem - Fluoroquinolone - Glycopeptide - Macrolide - Combinations of different classes • 3rd–4th cephalosporin generation + nitroimidazole • Carbapenem + glycopeptide • Other combinations ¶	26 (28) 30 (33) 14 (15) 4 (4) 1 (1) 1 (1) 16 (18) 5 3 8
Documentation of prior antimicrobial therapy, n (%) ^§^,	
- Empirical - Targeted • Catheter infection • Hemoculture • Urinary tract infection • Cholangitis • Respiratory tract infection	67 (73) 25 (27) 4 12 2 2 5
No. of days of previous antibiotic therapy, median (IQR)	7 (2–18)
No. of patients free of antibiotics > 7 days before drainage	31 (44)
CRP (mg/dl); day before drainage, mean [95% CI]	123 ± 104 [98–148]
Fever (T° > 38.5 °C); day before drainage, n (%)	28 (39.4)
WBC count; day before drainage (× 10^3^/mm^3^)	10.7 ± 6.3 [9.2–12.2]
Systemic inflammatory response syndrome, n (%)	21 (29.6)

CI: confidence interval; IQR: interquartile range; No: number; WBC: white blood cell. Quantitative data are expressed as mean ± standard deviation [CI 95%] unless otherwise indicated; ^†^ These data include the period between admission and the first intervention for infected pancreatic necrosis. ^‡^ Always used combined with antibiotics; ^§^ n = 92 antibiotic therapies administered to 56 patients; n = 1: penicillin + ß-lactamase inhibitors and glycopeptide; n = 1: antipseudomonal penicillin + ß-lactamase inhibitors and glycopeptide; n = 1: 2nd-generation cephalosporin and nitroimidazole; n = 1: fluoroquinolone and glycopeptide; n = 1: fluoroquinolone and nitroimidazole; n = 1: 3rd-generation cephalosporin, penicillin + ß-lactamase inhibitors, and nitroimidazole; n = 1: carbapenem and aminoglycoside; n = 1: carbapenem, glycopeptide, and lincosamide; ≥2 criteria out of the following: T° > 38 or T° < 36 °C, heart rate > 90 bpm, respiratory rate >20 per minute or Pa CO2 < 32 mmHHg, WBC > 12 103/mm^3^ or < 4 103/mm^3^; ¶ Confirmed (n = 52) and suspected (n = 2) IPN.

**Table 3:** Characteristics of antimicrobial and antifungal therapy after 1st intervention.

Characteristic	WON Acute Pancreatitis Cohort (N = 71)
No. of patients with IPN (confirmed/suspected), n (%)	52 (73%)/2 (3%)
No. of patients with antibiotic after 1st intervention, n (%)	70 (99)
Type of antibiotic therapy after the first intervention, n (%) ^†^	
- Penicillin + ß-lactamase inhibitors - Antipseudomonal penicillin + ß-lactamase inhibitors - Carbapenems - 1st- and 2nd-generation cephalosporin - Combinations of different classes • 3rd-generation cephalosporin and nitroimidazole • Carbapenems and nitroimidazole • Other combinations ^‡^	24 (34) 22 (32) 12 (17) 2 (3) 10 (14) 2 2 6
Accuracy of 1st antibiotic based on 1st bacteriological sample, n (%)	51 (73)
Antifungal therapy, n (%) ^§^	31 (44)
No. of antibiotic adaptations per patient, median IQR	2 [1–4]
No. of different antimicrobial therapies per patient, median (IQR)	4 [2–6]
Total duration of antibiotic therapy, days, [95% CI]	43 ± 30 [36–51]

IQR: interquartile range; No: number; Quantitative data are expressed as mean ± SD [95% CI] unless otherwise indicated; ^†^ n = 70 ^‡^ n = 1: fluoroquinolone and nitroimidazole; n = 1: carbapenem and glycopeptide; n = 1: carbapenem, penicillin + ß-lactamase inhibitors, and nitroimidazole; n = 1: carbapenem, fluoroquinolone, and glycopeptide; n = 1: penicillin + ß-lactamase inhibitors, carbapenem, and polymyxin; n = 1: 3rd-generation cephalosporin, nitroimidazole, polymyxin, and cotrimoxazole. ^§^ always used combined with antibiotics.

**Table 4:** Factors associated with the emergence of multidrug-resistant (MDR) bacteria after all interventions.

	Univariate Analysis			Multivariable Analysis	
Variable	OR [95% CI]	*p*-Value	Variable	b [95% CI]	*p*-Value
Age	0.99 [0.96–1.02]	0.642			
Prior exposure to ATB	3.67 [1.11–14.54]	**0.040**			
Changes in ATB	1.82 [1.34–2.64]	**0.0005**	Changes in ATB	1.70 [1.18–2.43]	**0.004**
ICU admission	4.44 [1.63–12.96]	**0.0046**			
Size collection (length)	1.01 [1.0–1.02]	**0.0466**			
Size collection (width)	1.01 [1–1.02]	0.058			
Type 2 diabetes status	1.69 [0.60–4.97]	0.329			
PPI	5.83 [0.87–115.0]	0.116			
BMI >30	1.36 [0.49–3.83]	0.550			
Nutritional support	8.27 [2.76–28.9]	**0.0003**	Nutritional support	5.69 [1.52–20.50]	**0.010**
AP duration	1.04 [1.02–1.06]	**0.0004**			
Albumin (g/L)	0.92 [0.84–0.99]	**0.027**			
Prealbumin (mg/L)	0.95 [0.86–1.04]	0.284			
Number of necrosectomies ≥ 3	4.71 [1.07–32.96]	0.062			
Number of total procedures ≥ 5	8.4 [2.81–29.31]	**0.0003**			

AP: acute pancreatitis; ATB: antibiotic; ICU: intensive care unit; BMI: body mass index; PPI: proton-pump inhibitors; OR: odds ratio; CI: confidence interval; MDR: multidrug-resistant. Bold values indicate statistically significant results (*p* < 0.05).

**Table 5:** Factors associated with fungal infections at the first intervention.

	Univariate Analysis	
Variable	OR [95% CI]	*p*-Value
Age	1.03 [0.99–1.06]	0.135
Prior exposure to ATB	1.20 [0.35–4.81]	0.781
Changes in ATB	1.17 [0.91–1.51]	0.219
ICU admission	2.69 [0.88–9.37]	0.096
Size collection (length)	1.00 [0.99–1.01]	0.591
Size collection (width)	1.01 [1.00–1.02]	0.192
Type 2 diabetes status	1.95 [0.64–5.84]	0.232
PPI	0.83 [0.15–6.32]	0.833
BMI >30	0.80 [0.25–2.39]	0.696
Nutritional support	3.08 [0.97–11.89]	0.051
AP duration	1.00 [0.99–1.01]	0.459
Albumin (g/L)	0.93 [0.85–1.01]	0.111
Prealbumin (mg/L)	0.97 [0.87–1.07]	0.563
Number of necrosectomies ≥ 3	2.81 [0.70–11.40]	0.138
Number of total procedures ≥ 5	3.43 [1.20–10.22]	**0.023**

AP: acute pancreatitis; ATB: antibiotic; ICU: intensive care unit; BMI: body mass index; PPI: proton-pump inhibitors; OR: odds ratio; CI: confidence interval. Bold values indicate statistically significant results (*p* < 0.05).

**Table 6:** Factors associated with the occurrence of IPN in general.

	Univariate Analysis			Multivariable Analysis	
Variable	OR [95% CI]	*p*-Value	Variable	b [95% CI]	*p*-Value
Age	1.02 [0.99–1.06]	0.198			
Prior exposure to ATB	9.00 [2.59–34.27]	**0.0007**	Prior exposure to ATB	26.41 [1.90–366.38]	**0.015**
Changes in ATB	1.60 [1.14–2.41]	**0.014**			
ICU admission	4.80 [1.52–17.21]	**0.010**			
Size collection (length)	1.00 [0.99–1.01]	0.823			
Size collection (width)	1.01 [1–1.02]	0.160			
Type 2 diabetes status	0.92 [0.29–3.30]	0.896			
PPI	8.00 [1.41–62.45]	**0.024**			
BMI >30	1.17 [0.37–4.15]	0.801			
Nutritional support	3.71 [1.21–12.02]	**0.023**			
AP duration					
Albumine (g/L)	0.76 [0.66–0.86]	**<0.0001**			
Prealbumin (mg/L)	0.83 [0.71–0.93]	**0.0035**	Prealbumin (mg/L)	0.77 [0.63–0.94]	**0.012**
Number of necrosectomies ≥ 3	0.72 [0.62–0.84]	0.052			
Number of total procedures ≥ 5	3.46 [0.99–16.29]	0.073			

AP: acute pancreatitis; ATB: antibiotic; BMI: body mass index; ICU: intensive care unit; IPN: Infected pancreatic necrosis; PPI: proton-pump inhibitors; OR: odds ratio; CI: confidence interval. Bold values indicate statistically significant results (*p* < 0.05).

## Data Availability

The data supporting the results of the present study are available from the corresponding author upon request.

## Supplementary Materials

The following supporting information can be downloaded at https://www.mdpi.com/article/10.3390/antibiotics15020220/s1, Supplementary Table S1: Data collected for each patient for this study. Supplementary Table S2: Microbiological data collected for each patient as well as definitions regarding in vitro antimicrobial susceptibility testing. Supplementary Table S3: The different microbiological agents isolated from the sample during the first intervention.
