# Supplementary material for: Risk Factors Associated with the Emergence of Multidrug-Resistant Bacteria and Fungal Infections in Walled-Off Pancreatic Necrosis
Source: Antibiotics (Basel). 2026 Feb 17;15(2):220. doi: 10.3390/antibiotics15020220 (PMC12938492; doi:10.3390/antibiotics15020220)
Supplement: Supplementary file 1 [file antibiotics-15-00220-s001.zip › antibiotics-4140285-supplementary.pdf]

Supplementary Table S1: Data collected for each patient from the study population

**Demographics**	• Age<br>• Gender
**Co-morbidities**	• Diabetes<br>• BMI
**AP characteristics**	• Aetiology<br>• Primarily admitted or transferred patient<br>• Disease severity according to Atlanta classification<br>• Presence of transient (<48h) or persistent (>48h) organ failure<br>• SIRS (at least two criteria present)<br>• C-reactive protein and white blood cell count *<br>• Albumin and pre-albumin blood levels *<br>• CT severity index **<br>• extent of necrosis (<30%/30-50%/>50%)
**Therapeutic modalities (apart antibiotic therapy and interventions)**	• Use of proton-pump inhibitor therapy<br>• Need for nutritional support (enteral or parenteral nutrition)
**Interventions**	• Timing between onset of symptoms and the first intervention<br>• Type and number of interventions (e.g., endoscopic, percutaneous, surgical)
**Outcome**	• Admission to ICU

	• Length of ICU stay<br>• Length of overall stay<br>• Mortality

BMI: Body mass index, ICU: Intensive care unit, SIRS: systemic inflammatory response syndrome
* day before first intervention
** after a minimum of 48-72h from onset of symptoms

Supplementary Table S2: **Microbiological data collected for each patient from the study population**

**Antibiotic use**	• Prior exposure to antibiotics or antifungal therapy before intervention<br>• type of therapy (empirical/targeted)<br>• type of antimicrobial agent before and after the intervention *<br>• duration of antibiotic/antifungal therapy<br>• Number of changes
**Cultures**	• Results of bacteriological and fungal cultures (WON fluid, blood)<br>• MDR, XDR and PDR status **

* Classified as: penicillin + ß lactamase inhibitors, antipseudomonal penicillin + ß lactamase inhibitors, 1st and 2nd generation cephalosporins, 3rd and 4th generation cephalosporins, carbapenems, fluoroquinolones, glycopeptides, 5-nitroimidazole, macrolides, and others (oxazolidinone, glycylcycline, aminoglycosides, polymyxins, monobactams, lincosamides, and cotrimoxazole). Data for combinations of different types of antimicrobial agents were also collected.

** MDR: multidrug-resistant, XDR: extensively drug-resistant, PDR: pan drug-resistant
According to in vitro antimicrobial susceptibility testing, each microbe was classified as: Non-MDR: non-susceptible to <1 agent in <3 antimicrobial categories, MDR: non-susceptible to ≥1 agent in ≥3 antimicrobial categories, XDR: non-susceptible to ≥1 agent in all but ≤2 categories, or PDR: non-susceptible to all antimicrobial agents listed
In cases where multiple resistant bacteria appeared during the subsequent interventions, the emergence of resistance was assessed according to the most resistant strain detected

WON: Walled-off necrosis

Supplementary Table S3: **Bacteria and fungi identified in the sample during the first intervention**

Bacteria, n (%)	*N=76*
Aerobes (N=71)	
- *Gram-negative bacilli*	*N=35 (49%)*
Acinetobacter baumanii	1 (1.4)
Burkholderia dolosa	1 (1.4)
Citrobacter freundi	1(1.4)
Escherichia Coli	**16 (22.5)**
Enterobacter aerogenes	2 (2.8)
Enterobacter cloacae complex	2 (2.8)
Enterobacteria sp	1 (1.4)
Hafnia alvei	1 (1.4)
Klebsiella pneumoniae	**5 (7)**
Morganella morganii	1 (1.4)
Proteus mirabilis	1 (1.4)
Pseudomonas aeruginosa	**3 (4.2)**
- *Gram-positive Cocci*	*N=32 (45%)*
Coagulase-negative staphylococcus	**7 (9.9)**
Enterococcus sp	1 (1.4)
Enterococcus faecium	**6 (8.5)**
Enterococcus faecalis	**4 (5.6)**
Enterococcus rafinosus	2 (2.8)
Staphylococcus epidermidis	2(2.8)
Staphylococcus haemolyticus	2 (2.8)
Streptococcus anginosus	1 (1.4)
Streptococcus constellatus	1 (1.4)
Streptococcus mitis	1 (1.4)
Streptococcus parasanguis	1 (1.4)
Streptococcus vestibularis	1 (1.4)
Streptococcus viridans	3 (4.2)
- *Gram-positive bacilli*	*N=3 (3%)*
Micrococcus luteus	1 (1.4)
Propionibacterium acnes	1 (1.4)
- *Aerobic Bacteria not identified, n (%)*	2 (2.8)
Anaerobes (n=5)	
Fusobacterium nucleatum	2 (40)
Lactobacillus gasseri	1 (20)
Prevotella buccae	1 (20)
Veillonella parvula	1 (20)
Fungi, n (%) (n=21)	
**Candida albicans**	**17 (81)**
Candida glabrata	3 (14.3)
Candida krusei	1 (4.7)

IPN: Infected pancreatic necrosis, Categorical data are expressed as n (%)
Microorganisms highlighted in bold represent the most frequently identified pathogens within each category.
